# An Exploration of Perceived Stress, Burnout Syndrome, and Self-Efficacy in a Group of Polish Air Traffic Controllers and Maritime Navigators: Similarities and Differences

**DOI:** 10.3390/ijerph18010053

**Published:** 2020-12-23

**Authors:** Marta Makara-Studzińska, Maciej Załuski, Paweł Jagielski, Dorota Wójcik-Małek, Michał Szelepajło

**Affiliations:** 1 Department of Health Psychology, Faculty of Health Sciences, Collegium Medicum Jagiellonian University, 31-008 Kraków, Poland; marta.makara-studzinska@uj.edu.pl (M.M.-S.); paweljan.jagielski@uj.edu.pl (P.J.); 2 Toxicology and Internal Ward, Ludwik Rydygier Hospital, 31-826 Kraków, Poland; dorotaannawojcik@gmail.com; 3 Department of Psychiatry, Pomeranian Medical University, 70-204 Szczecin, Poland; michal.szelepajlo@pum.edu.pl

**Keywords:** perceived stress, burnout syndrome, air traffic controllers, maritime navigators, self-efficacy, men

## Abstract

Background: This cross-sectional study aimed to assess the level of perceived stress and occupational burnout in groups of Polish maritime navigators and air traffic controllers. The study was part of research into occupational groups regarded as equally emotionally burdened. We tested the usability of a model linking occupational burnout, perceived stress, and seniority. Methods: The set of questionnaires, including the link burnout questionnaire, perceived stress scale—10, and generalized self-efficacy scale were distributed to 54 maritime navigators and 88 air traffic controllers (rate of return: 18–56%). Spearman’s rho, χ^2^ test, the Mann–Whitney U test, Cohen’s d and Hedge’s g coefficients, linear regression, and F statistic were used. Results: The assumption that persons employed in occupations with the special professional requirements as air traffic controllers and maritime navigator with a risk of strong, chronic emotional overload evaluate their life situation as less stressful than other employees was confirmed. A higher level of occupational burnout was observed in groups of controllers and navigators compared to an equally emotionally burdening occupational group of Polish firefighters, but not Polish psychiatrists. The research groups differed regarding the source of stress: fear of helplessness in the air traffic controller group and inefficacy in overcoming adversities in the maritime navigators. Maritime navigators reported a higher level of occupational burnout, deterioration of relations with coworkers, and disappointment with their work compared to the air traffic controllers. Conclusions: The results showed differences in factors linked to workplace demands and the personal predispositions of employees, and the role these may play in mutual relations between occupational burnout, life situation evaluation, and personal resources. We postulate that the level of perceived stress should be taken into account in the studies of occupational burnout syndrome.

## 1. Introduction

In line with the assumptions of the occupational burnout model: job demands—resources (JD-R) [1], each professional environment has its own factors influencing employees’ well-being and job satisfaction. Professional work engages two processes in a person: 1. energy, which is responsible for enhancing experienced stress and leads to a depletion of psychophysical resources and deterioration of health; 2. motivation, which affects the employees’ level of commitment and work satisfaction. Occupational burnout syndrome is defined as a psychosomatic state of a working person characterized by a cynical attitude towards work values as well as no hope for effective working performance. This is a result of depletion of mental and psychical energy as well as the cognitive resources of a person [2]. The symptoms of occupational burnout develop as a consequence of increases in overload caused by workplace requirements until a person’s psychophysical resources are depleted which decreases their motivation to engage with their work. Work and personal resources, understood as a person’s traits or state of mind, including their convictions on having control and influence over their work environment, have an important role in the phenomenon described above. These personal resources balance the requirements of the work environment and replenish the employee’s psychophysical reservoir. Xanthopoulou et al. [3] researched the role of three personal resources: generalized self-efficiency, self-esteem, and optimism, in predicting occupational burnout and changes in work commitment. Researchers noticed that personal resources partially mediated the relationship between work resources and commitment to work, protecting from burnout. Therefore, it is believed that positive self-esteem and a sense of control over the environment can alleviate the negative effect of work environment demands and simultaneously increase an employee’s commitment [4]. According to social cognitive theory, stress reactions depend on self-appraisals of coping capabilities [4]. Self-efficacy is defined as one’s belief in the possibility of achieving an intended goal, belief that one is able to carry out a certain activity and get a successful result. The high level of self-efficacy supports the ability to cope in a given situation at work and positively cope with stress [5]. It is a personal resource supporting demanding actions that require persistence and are characterized by a high level of complexity [5]. Prati et al. [6] observed that self-efficacy is an important resource in rescue workers. Scientific knowledge confirms the correlation between occupational burnout and chronic work-related stress [1,7]. According to the transactional model of stress, psychological stress is a result of the transaction between the individual and the environment, when environment demands are evaluated as exceeding the individual resources, especially in personally significant situations [8] (pp. 16–17). The impact of stressful events is, to some degree, determined by one’s perception of their stressfulness, in categories of their predictability, control, and sources of overload [9]. Psychological stress is influenced by both the objective features of a situation (i.e., work requires constant cognitive, emotional, or physical effort) and dispositional variables, including the aforementioned sense of self-efficacy and a subjective evaluation of efforts undertaken during struggles with daily adversities.

There is a group of professions that require from an employee specific psychological predispositions, for example, a specific cognitive competence called situational awareness (SA). This competence allows the employee to perceive elements of the work environment in space and time in an optimal way and properly understand the meaning of information in the present moment and the near future [10].

The group of professions that require specific personal predispositions includes air traffic controllers and maritime navigators. Although only 5% of commercial aircraft accidents resulted from air traffic controller errors, 55% of accidents that resulted from pilot errors, either directly or indirectly involved air traffic controllers. The analysis of the causes of all U.S. civil aviation accidents between 1985 and 1997 showed that the largest percentage of accidents related to air traffic controllers was skill-based error, indicating a breakdown in attention or memory processes of controllers, decision errors, and perceptual errors [11]. The consequence of the controllers’ error was, inter alia, the death of all 128 passengers in US airspace and 71 passengers of airplanes controlled by Swiss air traffic controllers [12]. Nearly half of all fatalities occurred during final approach and landing. The taxiing, climbing, approach, and landing are seen as critical safety factors of flight, because of the cooperation between a pilot and a controller [13]. Air traffic control has been classified as the fourth most stressful job, but according to Vogt and Kastner [14] explaining the job stress level of controllers only by the level of air traffic activity and potential conflicts may be shortsighted. The personal resources like self-efficacy, organizational-based self-esteem, and the abilities to perceive and regulate emotions are antecedents of work engagement. The controller has to prioritize different tasks, manage their cognitive resources, and evaluate and control their performance continuously [15,16]. The human-factor causes can be attributed to 70–80% of accidents in high-hazard industries [17]. Physical fatigue and mental state belong to the five most important human factors affecting air traffic control performance [18]. Research indicates that psychological factors like neuroticism reduce cognitive processing capacity [19]. The controllers with a high neuroticism value could not manage a higher number of aircraft which resulted in more collisions, in an experimental study [20]. Mental workload, fatigue, stress, situation awareness, and decision making are human factors that influence a person’s performance and result in job errors [21]. The decision-making process of air traffic controllers who are chronically overloaded by workplace demands which exceed their resources may lead to a series of negative consequences. The most important are health deterioration, symptoms of occupational burnout syndrome, and the occurrence of risky situations at work. In the case of air traffic controllers, there is a possibility of “loss of picture”, which is the correct link between the idea of an aircraft’s position in the controller’s mind and its actual position. Just as many mistakes are made in situations of underload, which raises the role of the optimal stress level for cognitive function efficiency [22]. The so-called complacency syndrome—a sense of self-satisfaction accompanied by unawareness of actual dangers—may explain the causes of air accidents occurring during routine flights [23]. The results of the study showed that high-stress events kept the controllers reacting in terms of both their cognitive and hormonal responses that significantly affected their performance [24]. The errors in the subjective assessment of the stress level and assessment of the drop in the efficiency of cognitive processes were revealed. An increased level of psychological stress impairs cognitive skills such as attention and decision making of air traffic controllers [22].

According to Endsley [25], changes in the organization of pilots’ and traffic controllers’ work, new technologies, and automatization may lead to controllers’ work becoming more passive, which may lead to a loss of optimal efficiency in cognitive functions and abilities and increase work overload, while a high level of acute stress may lead to overconfidence and more risky behaviors. A similar situation takes place at sea. Research has shown that in 48% of risky behaviors by deck officers driven by stress, the intermediary factor was a negative change in cognitive function efficiency [17]. Much research indicates that sailors are the occupational group most exposed to occupational stress [26]. This is also the group in which a strong connection between the occurrence of chronic occupational stress and the number of accidents was observed [27]. For this reason, the next generations of crew resource management (CRM) include new error management systems applied in high-risk environments: air traffic control [23,28] and maritime [29]. Their role is, among others, implementing strategies for self-detection of errors, explaining their sources and correcting them, and identifying personal factors contributing to errors. Among them are mentioned: coping with uncertainty, awareness of vulnerability to errors and accepting the possibility of errors, coping with frustrations from errors, avoidance of self-criticism and dwelling on the history of the error, and improving the vigilant attitude to counteract complacency [30].

Also, in the profession of the maritime navigator, personal factors have contributed to the formation of dangerous situations at sea. More than 80% of marine accidents occur due to errors caused by navigators. The fear appearing in the last moments of a collision encounter leads the navigator to the failure to perform prescribed collision-avoidance measures. If ship operators demonstrate high perceived collision risk resulting from fear of collision, they may panic and become less likely to avoid collision causing a marine accident [31]. A review of 100 shipping incidents that considered the cognitive demands, as the mental workload increased, the threat of a collision increased as well [31]. Between 2004 and 2013, 1328 accidents involving passenger and cargo ships took place in the Baltic Sea. The largest share of these accidents was grounding (36%) and collisions (29%). The most common cause of these accidents was human error (28%) [32]. Therefore, evaluating the human characteristics of the navigator as a source of human error is necessary to prevent future collisions. Accident analyses from the offshore oil-drilling industry showed that 67% of human errors can be attributed to a lack of perception of critical signals, 20% can be attributed to comprehension, and 13% to the inability to project the status of the situation in the near future [17]. The low-stress tolerance could result in a poorer ability to detect critical signals and perceive and anticipate them, which are the core elements of SA. A low level of neuroticism and a high level of extraversion and conscientiousness (resilient personality type) co-occurred with a high level of situation awareness. A type of approach to a risky situation, the so-called stimulus risk vs. instrumental risks and personality traits may determine the number of navigational errors as well [33]. There are several methods of analyzing human errors that occur during maritime accidents. Those are methods of human reliability analysis, cognitive reliability and error analysis, and human error reduction technique [30].

There are known stressors occurring in the workplace of sailors and air traffic controllers [27,34,35,36,37]. In a group of seafarers, a number of sources of job stress include deprivation of a number of basic needs resulting from the prolonged separation from family and friends, organizational stressors (the watch system, shortage of time for sleep, night shifts), physical stressors (rocking, vibrations, noise, changes in climate and time zones), and social and psychological stressors (the tension resulting from being in a closed group of people, the perception of constant emotional tension throughout the period of the cruise, fear of collision, responsibility for people and equipment). In a group of air traffic controllers, a number of sources of job stress includes workload and time pressure. The work environment requires employees to have capability to make quick and assertive decisions, perform physical assessments, capability to perform complex tasks with strict proficiency, responsibility for the safety of aircraft and their passengers, making decisions under time pressure, making multiple safety decisions in quick succession. There are no scientific reports on the intensity of stress perceived by the aforementioned occupational groups and occupational burnout and a feeling of self-efficacy. 

The results presented in this article are part of the research conducted in Poland in order to assess the level of perceived stress and burnout syndrome in professions characterized by the presence of high job demands. The first purpose of the present study was to compare the levels of perceived stress and burnout syndrome among groups of air traffic controllers, maritime navigators, firefighters, and psychiatrists from Poland. Due to the special professional requirements faced by air traffic controllers, on which the lives of many people may depend, and maritime navigators responsible for property of great value, environmental protection, and the life of seafarers, we assumed that the above-mentioned professional groups should employ people with special personal predispositions. Therefore, the level of perceived stress in the groups of controllers and navigators should be lower than in the groups of firefighters and psychiatrists. Both professional groups are characterized by high levels of stress and the risk of burnout syndrome [38,39]. The second purpose was to test the assumptions about the relationship between the level of perceived stress and burnout syndrome in both groups. According to the job demands–resources model of occupational burnout [1] and the role of personal resources [3], we expected that high levels of stress would be associated with high levels of burnout. The third purpose was to check the role of the individual differences in the appraisal of perceived stress, in the appraisal of self-efficacy and seniority in explaining the causes of burnout in the professions of air traffic controllers and maritime navigators. The relationship between self-efficacy and burnout is well known [3], but in the vast majority of scientific studies about the relationship between job stress and occupational burnout, the severity of job stressors is examined. There are no studies in which stress is understood as the result of a subjective employee’s appraisal of the situation. This approach takes into account the role of personal predispositions in the formation of a stress transaction. There are conflicting data from studies on the relationship between seniority and burnout syndrome.

The research goal was to verify the following hypothesis:

1. The groups of air traffic controllers and maritime navigators appraise the situations of lives as less stressful compared to the group of Polish firefighters and psychiatrists;
2. The perception of stress as a subjective evaluation of experienced life event is an important psychological risk factor for occupational burnout in the groups of air traffic controllers and maritime navigators;
3. Both the level of self-efficacy and the appraisal of the situations of lives as stressful shape the level of burnout in the occupations of air traffic controllers and maritime navigators. The age of life and seniority do not explain the causes of the occupational burnout syndrome.

## 2. Materials and Methods

### 2.1. Subject

The survey was conducted in the period from January to May 2020. In the case of the group of air traffic controllers, questionnaires were sent to all controllers working in 18 air traffic control centers of the Polska Agencja Żeglugi Powietrznej (PANSA) (Polish Air Navigation Services Agency). In the case of maritime navigators, the sampling method was used and questionnaires were sent to participants of two courses for promotion to the position of Chief Officer and Master Mariner, organized by the Training Center for Marine Officers at the Maritime University of Szczecin. 

The sociodemographic characteristic of the air traffic controllers and maritime navigators is shown in Table 1. The groups differ significantly regarding age: men over 36-years-old prevailed in the air traffic controller group, over 35-years-old in the navigators group. Shorter seniority is considered as one of the burnout factors. Age and seniority were positively related to burnout syndrome in a group of 109 Italian air traffic controllers [39]. There are also results showing no relationship between occupational burnout, age, and seniority in the group of controllers [40].

Regarding marital status, the air traffic controller group had more single and remarried men, while the navigators group had more men living in informal relationships. The groups were not different regarding seniority, number of children, or having active hobbies.

### 2.2. Applied Research Tools

To evaluate the level of occupational burnout, the Polish language version of the link burnout questionnaire (LBQ) was used [41]. The questionnaire consists of 24 items that measure four dimensions of burnout: psychophysical exhaustion (PE), relationship deterioration (RD), feeling of occupational inefficacy (PI), and disappointment with performed work (DI). A complimentary indicator is the comprehensive index of burnout (OBS), which is the sum of all points gained in the test. The responders, using a Likert-type six points scale, evaluated 24 items using the following categories: 1—never, 2—seldom, 3—once or more times a month, 4—less than once a week, 5—a few times a week, 6—every day. In the standardized Polish sample for the group of uniformed services, the Cronbach’s α coefficients were: PE 0.81; RD 0.73; PI 0.56; and DI 0.85, in our study: PE 0.797; RD 0.655; PI 0.582, DI 0.845. The questionnaire for perceived stress scale—10 (PSS-10) is the tool most often used to measure psychological stress. The Polish version of the scale that was used in this study was developed by Juczyński and Ogińska-Bulik [42]. The results indicate an evaluation of the efficiency of strategies to cope with stress used by the subject. The examined person evaluates his or hers life events in categories of their predictability, control, and sources of overload. It consists of 10 questions to which the respondent answers using a five-choice Likert-type scale (0—never, 1—almost never, 3—quite often, 4—very often). The raw score is between 0 and 40 points. The number of points indicates an evaluation of the intensity of perceived stress. Cronbach’s α coefficient in a standardized Polish sample was 0.86, in our study it was 0.894. The Polish version of the generalized self-efficacy ccale (GSES) [43] was used to measure convictions on self-efficacy. The tool tests the strength of the subject’s conviction on their ability to cope with difficult situations and obstacles. Responders answered 10 questions using a four-choice Likert-type scale (1—no, 2—rather not, 3—rather yes, 4—yes). The score is between 10 and 40 points. The number indicates the strength of conviction. In the standardized Polish sample, Cronbach’s α coefficient was 0.85 and in our study it was 0.865.

### 2.3. Statistical Analysis

Statistical analysis of the results was carried out using PS IMAGO PRO 6 software (IBM SPSS Statistics 26, IBM, Armonk, NY, USA). The threshold of statistical significance adopted was <0.05. The assumption of a normal distribution of variables was verified by the Kolmogorov—Smirnov test. The distribution of results over measurements of perceived stress and four components of occupational burnout were right oblique, whereas the distribution of self-efficacy was left oblique. In order to check the significance of differences between groups χ^2^ and U, Mann—Whitney tests were used. Correlation analysis was carried out by calculating Spearman’s rank correlation coefficient. The coefficients Cohen’s d and Hedge’s g were used to evaluate the effect of size. To validate the hypothesis, the procedures of linear stepwise regression and F statistic were applied.

## 3. Results

The research covered data from 88 air traffic controllers and 54 maritime navigators. In the case of the group of air traffic controllers, 609 sets of questionnaires were sent, of which 340 were sent back (55.88%) and 110 were filled in properly (incomplete data was rejected). From these 110 sets, 88 from men were selected (22 questionnaires were filled by women). In the case of a group of maritime navigators, 100 questionnaires were sent, 56 were returned, each of them properly filled, and 54 questionnaires only filled by men (two questionnaires were filled by women) were selected for analysis. Before the analysis, the raw data were converted to “standardized units” for the sten scores using tables of standards. The firefighter comparison group consisted of 580 professional firefighters: all were men aged 20–58 years old (M _mean age_ = 35.26 years, SD = 6.74) and came from 12 Polish voivodeships [44]. The group of 57 psychiatry specialists from the different regions of Poland were 41 females and 16 males aged between 27 to 86 years (M = 47; SD = 12.23) [45]. Both studies used purposive sampling. 

The characteristics of the level of perceived stress, self-efficacy, and burnout syndrome in the sample of air traffic controllers and maritime navigators are presented in Table 2.

### 3.1. Level of Perceived Stress in the Research Groups

**Figure 1:**
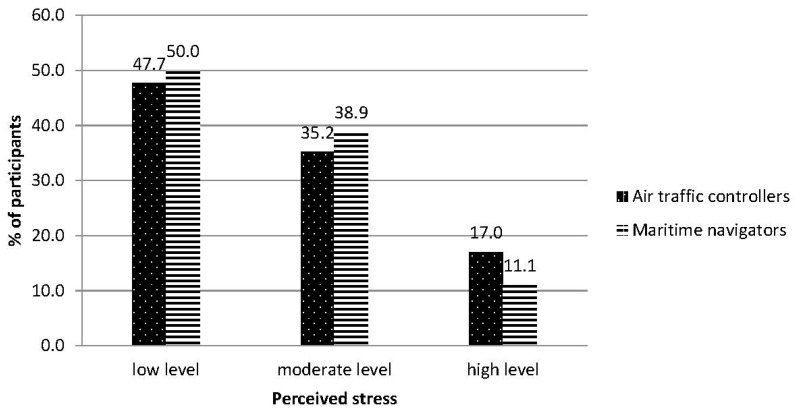
Levels of the perceived stress in both groups: air traffic controllers and maritime navigators. Note: The cut-off points low level ≤ 13 points; high level ≥20 points.

Both the air traffic controller group and the maritime navigator group showed low average results in the PSS—10 scale (respectively: 13.75 ± 6.89 and 12.78 ± 4.65) (see Table 2 and Figure 1). The median for the air traffic controller group was 14 points (range: 0–33 points), and the median in the maritime navigator group was 13.50 (range: 1–21 points). To compare the results of the evaluation of perceived stress, the conversion of raw data to standardized units was made using the sten scores, which were then grouped according to the rule: low result—1–4 sten scores (33% of observations), moderate result—5–6 sten scores (33% of observations), and high result—7–10 sten scores (33% of observations). The percentage share of low, moderate, and high results in both research groups is shown in Figure 1.

In each of the result ranges (low, moderate, and high), there were statistically insignificant differences between the tested groups (χ^2^ = 0.620). Low and moderate results prevailed in the maritime navigator group, and high results prevailed in the air traffic controller group. The data from persons from both groups were subjected to a factor analysis to check similarities within the structure of the tested variable. The subsequent measurement shows a two-factor structure of variables for data from the air traffic controller group [46]. Factor I, the so-called perceived helplessness, explained 49.029% of variances in the overall score, factor II, the so-called perceived self-efficacy—11.529%. In the case of data from the maritime navigator group, the analysis shows a three-factor structure of variables. Factor I explained 30.85% of variances, II—17.704, and III—11.46%. In this group, the factor with the greatest explanatory power was perceived self-efficacy, whereas the perceived helplessness factor was split into two, which were called: perceived nervousness and perceived control. The results of the air traffic controllers and maritime navigators were lower than in comparison to the groups of 580 Polish firefighters and 57 psychiatrists [44,45]. The result of firefighters was 14.86 ± 5.72 points. This is the result of the range of 5 sten scores. The Cohen’s d coefficient for the group of controllers and firefighters was 0.18 (Hedge’s g = 0.18); for the group of navigators and firefighters it was higher at 0.40. The result for the psychiatrists was 22.3 ± 8.34 points. This is the result of the range of 8 sten scores. The Cohen’s d coefficient for the group of controllers and psychiatrists was 1.12 (Hedge’s g = 1.14), and for the group navigators and psychiatrists it was 1.4 (Hedge’s g = 1.4).

### 3.2. Level of Occupational Burnout Syndrome in the Research Groups

**Figure 2:**
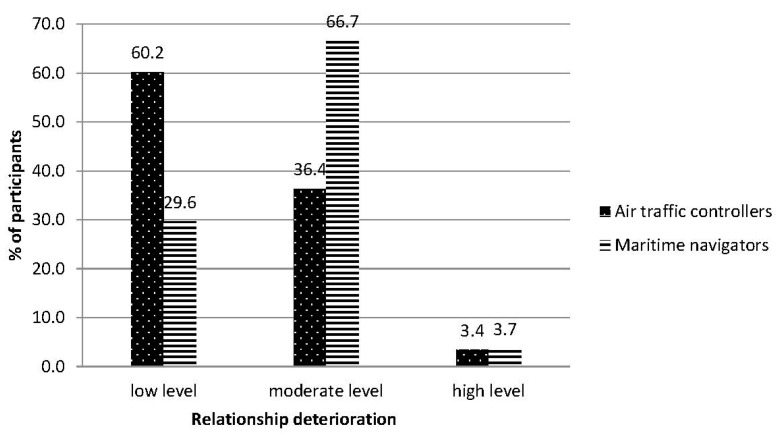
Levels of relationship deterioration in both groups: air traffic controllers and maritime navigators.

**Figure 3:**
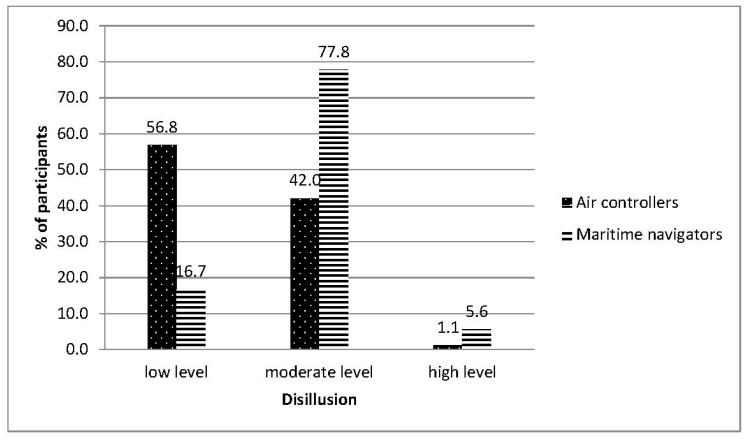
Levels of the disillusion in both groups: air traffic controllers and maritime navigators.

The measured level of occupational burnout in the groups of air traffic controllers and maritime navigators were in the range of 5 to 6 sten scores (moderate result). In terms of the severity of symptoms of occupational burnout research, the groups were statistically different (see Table 2). The summary index of occupational burnout calculations for the air traffic controller group was lower than that for the maritime navigator group. A large difference effect size occurred (Cohen’s d = 0.70; Hedge’s g = 0.69). Regarding the structure of burnout, the groups did not differ in levels of PE or feelings of professional inefficacy. Significant differences occurred regarding engagement in relations with coworkers/clients and disappointment with performed work. High values of difference effect were also observed (0.82 and 0.84, respectively; Hedge’s g = 0.79 and 0.83, respectively). The maritime navigators group results indicated a bigger severity of negative changes in the aforementioned two components of occupational burnout. In the air traffic controller group, low-level results prevailed, while in the maritime navigator group, moderate and high levels prevailed (see Figure 2 and Figure 3).

Regarding the burnout dimension PE, both groups had results noticeably higher than those of 580 Polish firefighters (M = 15.71 ± 5.61) [44]. The air traffic controllers showed an average value of different effect (Cohen’s d coefficient = 0.41; Hedge’s g = 0.43). In the case of the group of maritime navigator, the difference was even higher and amounted 0.66 (Cohen’s d coefficient) and 0.64 (Hedge’s g coefficient) [45]. The results obtained by 57 psychiatrists were as follows: PE = 20.46 ± 6.50; RD = 17.32 ± 4.44; PI: 17.05 ± 5.85; DI: 16.40 ± 6.12. The results of this group were significantly higher in all four dimensions of burnout syndrome compared to air traffic controllers and navigators. The Cohen’s d coefficients for the group of controllers and the group of psychiatrists were = 0.37; 0.71; 1.21; 0.84, respectively (Hedge’s g = 0.37; 0.69; 1.25; 0.84, respectively) [45]. The Cohen’s d coefficient for the group of navigators and the group of psychiatrists were = 0.20; 0.10; 1.11; 0.04, respectively (Hedge’s g = 0.19; 0.10; 1.01; 0.04, respectively). 

### 3.3. Level of Self-Efficacy in Research Groups

**Figure 4:**
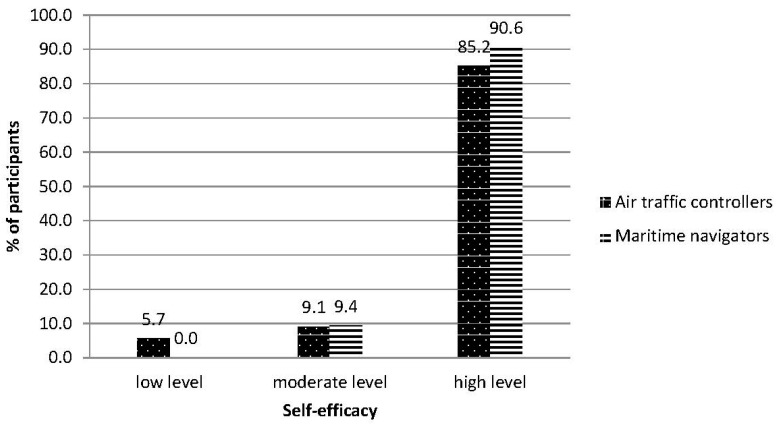
Levels of the self-efficacy in both groups: air traffic controllers and maritime navigators.

The averaged values of self-efficacy levels in the research groups were in the area of 8 sten scores, which is high. In the group of air traffic controllers, it was 33.57 ± 4.32 points and in the group of maritime navigators it was 32.84 ± 3.21 points. Levels of self-efficacy did not differ significantly between subjects from both groups. High levels of results prevailed in both groups; a slightly higher differentiation of results was observed in the air traffic controller group. Only in the air traffic controller group did subjects show low levels of the aforementioned variable (5%) (see Figure 4).

The results of both groups were noticeably higher than in the group of 580 Polish firefighters, who have obtained the average result = 31.89 ± 3.58. This is the result of the range of 7 sten scores. The Cohen’s d coefficient for the group of controllers and firefighters was 0.43 (Hegde’s g = 0.46), for the group of navigators and firefighters was 0.28 (Hedge’s g = 0.27). 

### 3.4. Correlations between Examined Variables

The coefficient values of the rho Spearman correlation, calculated separately for both examined groups, are shown in Table 3. The groups differed from each other. Moderately strong correlations between occupational burnout and levels of perceived stress and feelings of self-efficacy were observed in the air traffic controller group, whereas in the maritime navigator group, a correlation between occupational burnout and levels of perceived stress was not observed, only the feeling of self-efficacy.

### 3.5. Analysis of the Model Explaining Dependencies between Variables

The procedure for analyzing linear regression was performed on groups that included both professional groups in order to verify the correctness of the assumptions concerning the tested relations (hypothesis 3). Variables were subsequently introduced into the models: age, seniority, levels of perceived stress, and self-efficacy. A significant match of the assumed multivariate model was observed (F (4128) = 16.499, *p* < 0.0001). The model explained 34% of variances in the results of occupational burnout and included two relevant elements: levels of perceived stress and sense of self-efficacy (Table 4). 

## 4. Discussion

There are a small number of scientific reports on the results of levels of perceived stress in different occupational groups in the subject literature [47]. Most of the research concerns clinical populations, healthcare employees, and students. Groups of air traffic controllers and maritime navigators declared the result was lower than the values received from the survey on 1830 healthy Poles: 16.62 ± 7.50 (average age 36.7 ± 6.4) in standardized research on a PSS 10 questionnaire [42]. For comparison, the results of research conducted on a group of 28 American soldiers on active duty at an airbase in South Korea (14 women and 14 men) showed a correspondingly low stress level = 15.57 ± 7.04 [48]. In a study of 240 Chinese policewomen, the average level of perceived stress was 15.2 ± 5.6 [49]. In all the aforementioned cases, the results were low, yet higher than those obtained in our study. Results from some studies show the correlation of evaluation with the respondent’s sex and age [50]. There are correlations between the level of perceived stress and the number of critical live events in a group of younger people and lack of such a relationship in a group of older people [9]. Therefore, the research did confirm the assumption about low levels of perceived stress in groups of air traffic controllers and maritime navigators. Similar results were obtained in the study of Norwegian air traffic controllers [40]. This is good news from the perspective of the probability of making mistakes with tragic and costly consequences. It points to a compatibility of the personal predispositions of the tested groups with the expectations towards them. Regarding the results, it should be noted, however, that 17% of air traffic controllers and 11.1% of maritime navigators declared high levels of perceived stress in the one-month period preceding the test. Based on the information about differences in factor structure of the studied variable in both groups, it can be assumed that, in the case of air traffic controllers, the variability in the levels of perceived stress was determined mostly by feelings of perceived helplessness, and to a lesser extent, perceived self-efficacy, while the variability of levels of perceived stress in the maritime navigator group depended more on the feeling of perceived self-efficacy, and to a lesser extent on perceived nervousness and perceived control. The transactional theory of psychological stress states that a person can only declare they are experiencing stress when: 1. the situation is judged by a person as threatening their well-being or imposes essential requirements; 2. the person does not have sufficient resources to cope with the situation [9]. Therefore, the stress reaction does not depend only on the intensity of an event or its other characteristics, but mostly on dispositional and contextual factors, influencing the person’s evaluation. This evaluation determines at what level current life is perceived as unpredictable, uncontrollable, and overloaded. The tested air traffic controllers pointed out that the presence of signs of helplessness in life situations were the most significant sources of perceived stress, while the lack of efficiency in counteracting and controlling adversities had a lesser impact. In the case of maritime navigators, the evaluation of perceived stress was mostly influenced by feelings of self-inefficacy in overcoming adversities and, to a lesser extent, the feeling of general nervousness and lack of control over life events. The fear of helplessness in the air traffic controller group and the fear of efficacy in the maritime navigator group show the differences in the way the subjects evaluated stress. 

A higher level of occupational burnout was observed in groups of controllers and navigators compared to emotionally burdening occupational group of Polish firefighters, but not Polish psychiatrists. In order to find the causes of differences between the level of burnout syndrome in the groups of controllers and navigators and groups of firefighters and psychiatrists, the seniority variable was compared. Regarding the length of service, 58% of the air traffic controllers had worked from 11 to 20 years, compared with 50% of the maritime navigators, while among the firefighter group, those serving from 9 to 15 years prevailed at 41.32%, hence the firefighter group had a slightly shorter length of service. In terms of PE, the maritime navigators suffered the highest levels, followed by air traffic controllers, and then the firefighters. In RD, PI, and DI results, air traffic controllers came lower than the firefighters (respectively: 16.55 ± 4.40; 13.47 ± 4.39; 13.71 ± 6.13). In RD and DI results, the maritime navigators’ were higher than those obtained by the firefighters but in PR results came lower. Therefore, of the four dimensions of occupational burnout, the maritime navigators had results in three (PE, RD, and DI) worse than the air traffic controllers and firefighters. For comparison, the results obtained from a group of 30 Polish prison service officers having direct contact with inmates, which can also be assumed to be a very stressful job, were definitely lower in comparison to the three abovementioned occupational groups (PE = 7.03 ± 1.65; RD = 6.46 ± 2.06; PI = 6.13 ± 2.22; DI = 7.80 ± 1.21) [51].

The groups of air traffic controllers and maritime navigators did not differ significantly regarding feelings of self-sufficiency, which in the vast majority of cases was high. The results of the level of self-efficacy in both examined groups were noticeably higher than in the group of 34 Polish professional swimmers participating in regular training in the past six years (M = 30.45 ± 4.98, Cohen’s d = 0.67−0.57; Hedge’s g = 0.69−0.60) [44,45,52]. In comparative multicultural studies conducted on a group of 12,840 people, the average result was 28.63 ± 6.18 points, and higher results were observed in the group of men. The sample of Polish men obtained a result of 29.03 ± 4.70 points [43]. The aforementioned results were lower in the discussed groups. Subjects declaring a low level of self-efficacy (5.7%) were found in the air traffic controller group but not found in the maritime navigator group. Self-efficacy describes a belief in the ability to control difficult life demands and one’s responses to them. The intensity of the researched variable determines a person’s ability to control their actions in the most efficient way. However, high levels of self-efficacy, together with knowledge and ability only guarantee success when a person realistically evaluates a situation and their own abilities, otherwise, it may lead to risky decisions and behaviors, causing disappointment. A positive image of self-efficacy, if it is unrealistic, may become an internal stressor leading to occupational burnout. 

The research groups differed in terms of correlations between occupational burnout with perceived stress. The second hypothesis that high levels of perceived stress would be associated with high levels of burnout was partially confirmed. Moderately strong correlations between occupational burnout and levels of perceived stress were observed in the air traffic controller group, whereas in the maritime navigator group, a correlation between occupational burnout and levels of perceived stress was not observed. A positive correlation between these variables was observed in the validation research of the LBQ questionnaire, as well as in studies of Korean seafarers and Polish firefighters [44,53]. A higher level of occupational burnout corresponded with a higher level of perceived life stress. Similar correlations were observed in the air traffic controller group in which a positive correlation of moderate strength occurred. Only a moderately strong positive correlation between the level of perceived stress and one of the dimensions of occupational burnout—the loss of occupational efficiency—was observed in the maritime navigator group. The obtained result should be linked to the differences observed within the studied groups and considering the causes of the stress, which was mentioned previously in this article. The maritime navigator’s concerns about being inefficient in overcoming adversities were also the fundamental reason for evaluating life situations as stressful, and the effect of difficulties in objectively evaluating the results of their work as a result of occupational burnout. To a significant degree, perceived stress coexisted with all of the dimensions of occupational burnout, showing negative correlations of moderately high intensity in the air traffic controller group. Along with increased feelings of PE, feelings of occupational inefficiency and disappointment with performed work also increased and relations with coworkers got weaker. In the maritime navigator group, the increase in perceived stress decreased feelings of occupational efficiency, but it did not induce negative changes in other dimensions of occupational burnout. Correlations linking feelings of self-efficacy with stress and occupational burnout in both researched groups were different. As in the research of other authors, the beneficial influence of self-efficacy on levels of perceived stress was observed, which shows the intercorrelations of the aforementioned variables. Evaluations of life situations as controllable and predictable, and not being a source of excessive overloads depends on personality features such as positive self-esteem, high self-efficacy, and problem-oriented methods of coping with stress. The results of the regression analysis confirmed the third hypothesis. The assumed model linking occupational burnout with levels of perceived stress and feelings of self-efficacy turned out to be correct. The age of life and seniority did not explain the causes of the occupational burnout syndrome. Similar dependencies were observed in other occupational groups: Polish firefighters and doctors [19,27]. High levels of perceived stress coexisted with all the dimensions of occupational burnout, where the level of self-efficacy moderated the relationship between stress and burnout. It was the first study concerning the issues of perceived stress and occupational burnout in occupational groups of air traffic controllers and maritime navigators conducted in Poland.

## 5. Conclusions

The results obtained in the study revealed differences, connected to occupation factors in the mutual relations of occupational burnout, perceived stress, and personal resources. The assumption that persons employed in occupations with the special professional requirements as air traffic controllers and maritime navigator with a risk of strong, chronic emotional overload evaluate their life situation as less stressful than other employees was confirmed (first hypothesis). This implies that dispositional factors (ability to manage your cognitive resources and self-detection of errors, coping with uncertainty, awareness of vulnerability to errors and accepting the possibility of errors, coping with frustrations from errors, avoidance of self-criticism), have a greater role in generating stress. At the same time, this study shows that representatives of occupational groups evaluate their personal lives as generally controllable and predictable, and not exceedingly overwhelming. Occupational burnout (second hypothesis) was partially confirmed, and the higher levels of occupational burnout in the examined groups were observed compared with other occupations, although not in all dimensions. The maritime navigators reported a higher level of occupational burnout including greater deterioration in relations with coworkers/clients and greater disappointment with performed work compared with the air traffic controllers, despite the lack of differences in the range of ways of evaluating life events and self-efficacy. The level of occupational burnout syndrome in the controllers and navigators was higher than in the Polish firefighter group and lower than in the group of Polish psychiatrists. The hypothesis was that that high levels of perceived stress would be associated with high levels of burnout was confirmed only in the air traffic controller group. The study confirmed the third hypothesis regarding the correlations of levels of the perceived stress, occupational burnout, and self-efficacy. The negative influence of cognitive appraisal (perceived stress) on the appearance of all symptoms of occupational burnout was clearly shown in that case. Age and length of service did not explain the variability of occupational burnout in the research groups. The study shows the need to take into account dispositional factors, and the stressful factors linked to the specifics of the occupation in explaining the reasons for employees’ occupational burnout. We achieved partial confirmation of the assumption on the protective influence of personal resources, which is the feeling of self-efficacy, on the level of burnout. This influence on the air traffic controllers concerns all of its dimensions, while in the maritime navigator group it only influenced the feeling of self-inefficacy. It highlights the merit of enhancing employees’ realistic beliefs in self-efficacy in order to prevent occupational burnout, especially because, in the air traffic controller group the main source of anxiety on which the evaluation of stress levels depended concerned feelings of helplessness in difficult situations and adversities, while in the maritime navigator group, it was feelings of inefficacy in overcoming adversities. The low level of occupational burnout identified in air traffic controllers may be probably a result of considering candidates with specific personal characteristics at the stage of selecting for work. The assumptions of the JD-R model of occupational burnout indicate an important role for enhancing occupational and personal resources in optimizing professional work performance and preventing psychophysical depletion and loss of health. While making organizational changes to reduce sources of work overload, employers should also consider how to enhance their employees’ personal predispositions, especially since these factors strongly influence the quality of performed work and occupational burnout. The results of our study confirm the validity of creating employee improvement systems not only in terms of professional but also psychological skills related to coping with fear and responsibility, self-control of the efficiency of cognitive functions, making a number of decisions under time pressure, and the realistic assessment of competences. The ability to separate the non-professional stress from the one occurring in the workplace ensures the sustainment of cognitive function at the highest level. We postulate that the level of perceived stress should be taken into account in the studies of occupational burnout syndrome.

The authors of the research are aware of their limitations, mostly concerning the methodology used. It was a cross-sectional investigation which is characterized by a low level of accuracy and does not allow to determine cause-and-effect relationships. The questionnaires were sent to all professionally active air traffic controllers in Poland, whereas the group of maritime navigators was not chosen in a way representative of that occupational population, therefore, the compared groups were not of equal populations. The group of maritime navigators was small and convenience sampling was used, which can affect the generalizability of the findings. Self-description tools were used in the research, which has a risk of measurement errors (reporting errors, method variance errors). The effects of social and economic events that might have affected their scores were not investigated in the study. Questionnaire-based cross-sectional studies bear the risk of errors caused by gaining data only from one source and reversed causality. In order to eliminate these limitations, longitudinal study procedures and experimental methods based on objective ways of measuring variables should be used (blood pressure, stress and ectopic beats, and cholesterol level). 

## Figures and Tables

**Table 1:** Sociodemographic characteristics of the survey sample (*n* = 142).

		Frequency	%	Frequency	%	Significance
		Air Traffic Controllers *n* = 88 (62.0%)		Maritime Navigators *n* = 54 (38.0%)		
**Age (years)**	Total sample					U = 0.0045 ***
	25–31	14	15.9	13	24.1	
	32–35	15	17.0	18	33.3	
	36–40	23	26.1	11	20.4	
	41–45	15	17.0	8	14.8	
	46–50	6	6.8	0	0	
	51–60	11	12.5	4	7.4	
	over 60	4	4.5	0	0	
**Seniority**	(years)					U = 0.5408
	1–5	18	20.5	10	18.5	
	6–10	19	21.6	17	31.5	
	11–20	51	58.0	27	50.0	

Note: Nonparametric statistics were used: Mann–Whitney U-test;*** *p* < 0.001.

**Table 2:** Group differences between air traffic controllers (*n* = 88) and maritime navigators (*n* = 54).

	M *±* SD	M *±* SD	Significance Mann–Whitney U	Cohen’s d (Hedge’s d)
	**Air Traffic Controllers**	**Maritime Navigators**		
PSS-10	13.75 ± 6.89	12.78 ± 4.65	0.472	
PSS-10 PH	9.83 ± 4.71	8.87 ± 3.21	0.243	
PSS-10 PS	3.92 ± 2.81	3.91 ± 2.45	0.978	
GSES	33.58 ± 4.33	32.85 ± 3.22	0.091	
(LBQ)index	54.36 ± 16.07	64.93 ± 14.03	0.0001 ***	0.70 (0.69)
(LBQ)PE	18.13 ± 6.09	19.30 ± 5.27	0.146	
(LBQ)RD	13.91 ± 5.15	17.74 ± 4.14	0.0001 ***	0.82 (0.79)
(LBQ)PI	10.90 ± 4.11	11.70 ± 3.45	0.069	
(LBQ)DI	11.43 ± 5.77	16.19 ± 5.54	0.001 ***	0.84 (0.83)

Note: Variables were expressed as: M = mean ± SD (standard deviation), Cohen’s d—effects size, Hedge’s g—effect size. Nonparametric statistics were used: Mann–Whitney U test, χ^2^-test; *** *p* < 0.001. PSS-10—perceived stress scale, PSS-10 PH—perceived stress scale perceived helplessness, PSS-10 PS—perceived stress scale perceived self-efficacy, GSES—generalized self-efficacy scale, (LBQ) index—link burnout inventory composite index of occupational burnout syndrome, (LBQ) PE—psychophysical exhaustion, (LBQ) RD—relation deterioration, (LBQ) PI—professional inefficacy, (LBQ) DI—disappointment.

**Table 3:** Spearman correlation coefficients between the studied variables.

	**Air Traffic Controllers**						
	1	2	3	4	5	6	7
PSS-10	-						
GSES	−0.412 **	-					
(LBQ)index	0.627 **	−0.468 **	-				
(LBQ)PE	0.535 **	−0.339 **	0.780 **	-			
(LBQ)RD	0.338 **	−0.316 **	0.721 **	0.361 **	^-^		
(LBQ)PI	0.485 **	−0.384 **	0.520 **	0.246 *	0.243 *	-	
(LBQ)DI	0.523 **	−0.473 **	0.834 **	0.597 **	0.503 **	0.408 **	-
	**Maritime Navigators**						
PSS-10	-						
GSES	−0.334 *	-					
(LBQ)index	0.197	−0.317 *	-				
(LBQ)PE	0.205	−0.195	0.834 **	-			
(LBQ)RD	0.045	−0.191	0.769 **	0.460 **	^-^		
(LBQ)PI	0.461 **	−0.367 **	0.441 **	0.296 *	0.162	-	
(LBQ)DI	0.119	−0.234	0.856 **	0.698 **	0.580 **	0.184	-

Note: * *p* < 0.05, ** *p* < 0.001. 1: PSS-10—perceived stress scale, 2: GSES—generalized self-efficacy scale, 3: (LBQ)index—link burnout inventory composite index of occupational burnout syndrome, 4: (LBQ)PE—psychophysical exhaustion, 5: (LBQ) RD—relation deterioration, 6: (LBQ) PI—professional inefficacy, 7: (LBQ)DI—disappointment.

**Table 4:** Multiple linear regression results for variables: occupational burnout syndrome, perceived stress, self-efficacy, age, and seniority in the group of air traffic controllers.

Variable	β	95% CI	*p*
PSS-10	0.374	0.579–1.393	0.0001
GSES	−0.325	(−1.930)–(−0.682)	0.0001
Age	−0.050	(−0.447)–0.259	0.598
Seniority	0.041	−(−3.023)–4.731	0.933

Note: PSS-10—perceived stress scale, GSES—generalized self-efficacy scale.

## Data Availability

Data available in a publicly accessible repository.
